# Function and mechanism of long non-coding RNA Gm21284 in the development of hippocampal cholinergic neurons

**DOI:** 10.1186/s13578-019-0336-5

**Published:** 2019-08-29

**Authors:** Xiang Cheng, Haoming Li, Heyan Zhao, Wen Li, Jianbing Qin, Guohua Jin

**Affiliations:** 1 Department of Human Anatomy, Medical School of Nantong University, Nantong, China; 2 Medical School of Nantong University, Building 3, No. 19 Qixiu Road, Congchuan District, Room 325, Nantong, 226001 China

**Keywords:** lncRNA, Cholinergic neuron, NSCs, Development, Hippocampus

## Abstract

**Background:**

Increasing evidence has revealed that long non-coding RNAs (lncRNAs) play a pivotal role in the development of nervous system. Our previous studies have demonstrated that enhanced cholinergic neurogenesis occurs in the subgranular zone (SGZ) of the hippocampal dentate gyrus (DG) after cholinergic denervation, which is closely associated with the core transcription factor Lhx8. This study aimed to identify novel lncRNAs in a denervated hippocampal niche, which may affect cholinergic neurogenesis, and to explore the molecular mechanisms underlying cholinergic neurogenesis.

**Methods:**

The gene expression profiles of the denervated hippocampus were examined by microarray analysis, and targeted lncRNAs were filtered using bioinformatics analysis. The lncRNA Gm21284 was predicted to be associated with Lhx8. RT-PCR and FISH were used to observe the expression and localization of Gm21284 in vitro and in vivo. The interaction between Gm21284 and Lhx8 and miR-30e-3P was verified using the luciferase reporter gene assay. Cell proliferation and differentiation was observed to reveal the effects of Gm21284 in cholinergic neurogenesis.

**Results:**

Microarray analysis demonstrated 482 up-regulated and 135 down-regulated mRNAs, 125 up-regulated and 55 down-regulated lncRNAs, and 10 up-regulated and 3 down-regulated miRNAs in the denervated hippocampal niche. Overall, 32 lncRNAs were differentially expressed in the denervated hippocampal niche, which could interact with miR-30e-3p, miR-431, and miR-147. Among these 32 lncRNAs, Gm21284 and Adarb1 were identified after interleaving with lncRNAs in a co-expression network and WGCNA. Gm21284 was mainly located in the hippocampal DG. Furthermore, Gm21284-positive cells were considerably increased in the denervated hippocampus than in the normal side. EdU proliferation assay revealed that the proliferation of neural stem cells was repressed after the overexpression of Gm21284. Compared with the control group, the proportion of ChAT-positive cells increased at 7 days of differentiation of NSCs overexpressing Gm21284.

**Conclusion:**

Thus, Gm21284 functions as a competing endogenous RNA, which inhibits the proliferation of hippocampal NSCs and promotes their differentiation toward cholinergic neurons by inhibiting miR-30e-3P competitively.

## Introduction

The hippocampus within adult brains is crucial for the formation of certain types of memory, such as episodic and spatial memory [1]. The projective fibers of cholinergic neurons in the septum of the basal forebrain mainly pass through the dorsal fornix and may release acetylcholine as a transmitter to the hippocampus, which is closely associated with cognitive functions [2, 3]. Our previous study led to interesting conclusions that quiescent NSCs in the subgranular zone (SGZ) of the hippocampal dentate gyrus (DG) are activated, proliferated, and differentiated toward cholinergic neurogenesis after the projection fibers of cholinergic neurons in the basal forebrain are severed [4]. In recent years, several researchers have speculated that Lhx8, a member of the LIM homeobox gene family, is crucial in the development of cholinergic neurons [5, 6]. In the early stage of embryonic development, Lhx8 is specifically expressed in the medial ganglionic eminence (MGE); cholinergic neurogenesis occurs in ventral MGE [7]. Cholinergic neuronal precursors in Lhx8-deficient transgenic fetuses can survive; however, they fail to develop into cholinergic interneurons. In Lhx8-deficient transgenic mice fetuses, the cholinergic neurons in the medial septum nucleus (MS) and nucleus of the vertical limb of the diagonal band (VDB) of the basal forebrain were significantly reduced, whereas the basal large nuclei where the cholinergic fibers project to the cortex disappeared [8]. Furthermore, the in vivo overexpression of Lhx8 in the DG reportedly promotes the differentiation of NSCs located in SGZ into cholinergic neurons after decholinergic innervation, whereas the injection of shRNA-Lhx8 lentivirus down-regulates cholinergic neurogenesis in the DG [9, 10]. The expression of Lhx8 also revealed a significant correlation to the number of ChAT-positive cholinergic neurons in vitro [10]. Taken together, this suggests Lhx8 to be a core TF, which plays an important role in the development of cholinergic neurons in the hippocampus.

An increasing number of human and mouse lncRNAs have been identified as key regulators of a variety of cellular processes, including proliferation, apoptosis, and stress response [11]. Many lncRNAs are specifically expressed in the brain, and the discovery of their role in brain development is being investigated. Ramos and his colleagues identified and predicted over 12,000 novel lncRNAs in the subventricular zone of adult mice. The expression level of 17 and 64 non-coding RNAs were found to change during the fate selection process of neural progenitor cells (NPCS) and differentiation of NPCS into GABAergic neurons, respectively. Moreover, the expression level of 100 non-coding RNAs changed during glial differentiation. Further, cell types were demonstrated to be directly associated with the appearance of specific non-coding RNA sets, suggesting a causal relationship between cell differentiation and lncRNA regulation [12, 13]. Ng et al. [13] used a customized lncRNA chip to identify 35 lncRNAs that were up-regulated during the differentiation of human embryonic stem cells (ESCs) into neurons. One of the lncRNA RMSTs was found localized in the chromatin at the promoters of the important neuromodulators Sp8 and Dlx2. The RMST can also directly bind to SOX2 and the RNA-binding protein hnRNPA2/B1, suggesting that lncRNA regulates transcription factors via hnRNP recruitment to downstream target genes. The lncRNA Dali can bind to the neurogenic transcription factor POU3F3. Deletion of Dali in the N2A neuroblastoma cell line inhibited its differentiation into neurons. Genome-wide analysis has shown that Dali can bind > 1000 genes, many of which are involved in cell cycle regulation and neuronal function [14]. Mercer et al. [15] observed 849 lncRNAs in the adult mice brain, specific for different anatomical regions, cell types, and subcellular organellar distribution. For example, many lncRNAs display a significant specific distribution in the hippocampus, cortex, olfactory bulb, and cerebellum, as well as in the sub-regions of CA1, CA2, CA3, and the hippocampal DG. In the cerebellar Purkinje cells, the abundance of lncRNA expression in nuclei and cytoplasm was also significantly increased [16]. The recognition of these region-specific lncRNAs not only suggests their probable involvement in specific neural activities but also helps in understanding the molecular mechanisms within the neural network structure and functional connectivity between regions. To our knowledge, the role of lncRNA in the regeneration and development of cholinergic neurons, particularly whether the core transcription factor of cholinergic neuronal development is regulated by non-coding RNAs, has not been reported. Recent studies have shown that lncRNAs localized in the cytoplasm can be used as competing endogenous RNAs (ceRNAs), which use binding sites with related miRNAs to bind miRNAs competitively and exert adsorption elimination, thereby down-regulating these miRNAs in cells. The abundance of such miRNAs can be due to the post-transcriptional regulation of downstream target genes [17, 18].

Based on this, our study aimed to investigate the specific molecular mechanism of Lhx8 in cholinergic neurogenesis within the hippocampus and to identify the related specific lncRNAs binding to Lhx8.

## Results

### 1. Gene expression profile in the hippocampal niche after cholinergic denervation

**Fig. 1:**
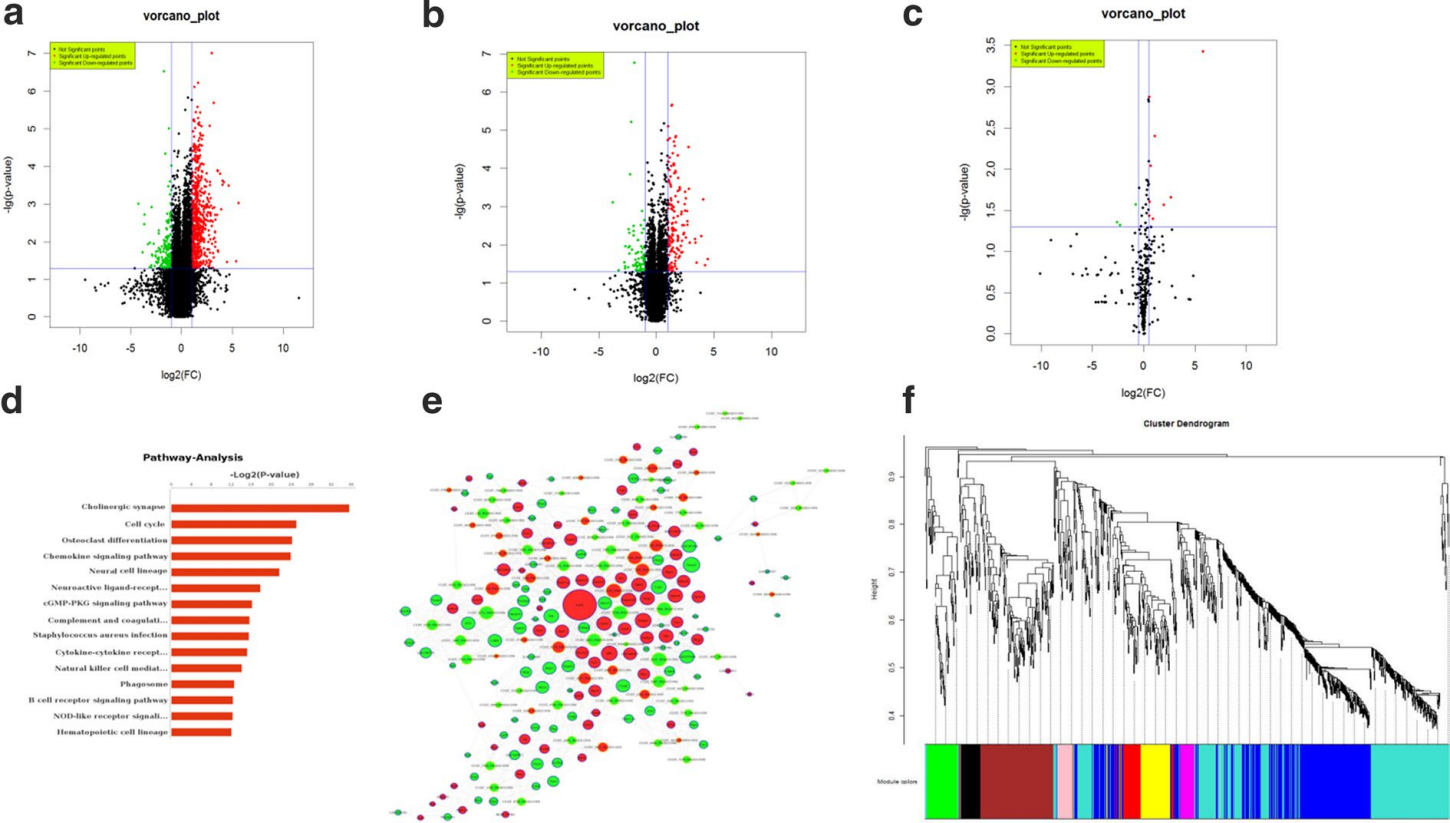
Differentially expressed transcripts in hippocampal niche at 7 days after fimbria–fornix transection. **a** The volcano map shows differently expressed mRNAs in hippocampal niche after fimbria–fornix transection, the screening threshold of differentially expressed genes (fold change) is ≧ 1.5, *P* < 0.05. **b** The volcano map shows differently expressed lncRNAs in hippocampal niche after fimbria–fornix transection, the screening threshold of differentially expressed genes (fold change) is ≧ 1.5, *P* < 0.05. **c** The volcano map shows differently expressed miRNAs in hippocampal niche after fimbria–fornix transection, the screening threshold of differentially expressed genes (fold change) is ≧ 1.5, *P* < 0.05. **d** Co-expression networks of differentially expressed genes in the transected group. Lhx8 is found in the network core and is associated with 111 mRNA and 26 lncRNAs. **e** Weighted co-expression network for differential genes in microarray detection. Lhx8 is present in the brown module

Gene expression analysis revealed 617 differentially expressed mRNAs in the hippocampal niche after cholinergic denervation. Among these, 482 mRNAs were up-regulated and 135 mRNAs were down-regulated in the transected group. Compared with the control group, the expression of Lhx8 in the transected group was up-regulated (2.45 ± 0.13-fold, *P *< 0.01). The differentially expressed genes are represented by a volcanic map (Fig. 1a). Microarray analysis identified 125 up-regulated and 55 down-regulated lncRNAs and 10 up-regulated and 3 down-regulated miRNAs in the denervated hippocampal niche (Fig. 1b, c) (Additional file 1). KEGG pathway enrichment analysis revealed that the differentially expressed mRNAs in the denervated hippocampal niche were mainly enriched in pathways involved in cholinergic synapse, neural development, cell cycle, and neural cell lineage (Fig. 1d). To explore the interaction between mRNAs and lncRNAs in a denervated hippocampal niche, we constructed a co-expression network of differential mRNAs and non-coding RNAs in the transected group based on the Pearson coefficient of each pair of genes. Overall, 137 genes, including 111 mRNA and 26 lncRNAs, were found to be associated with Lhx8 in the co-expression network (Fig. 1e). In WGCNA, Lhx8 was located in the brown module comprising 165 mRNA and 23 lncRNAs (Fig. 1f).

### 2. ceRNA for identification of differential lncRNAs associated with Lhx8

**Fig. 2:**
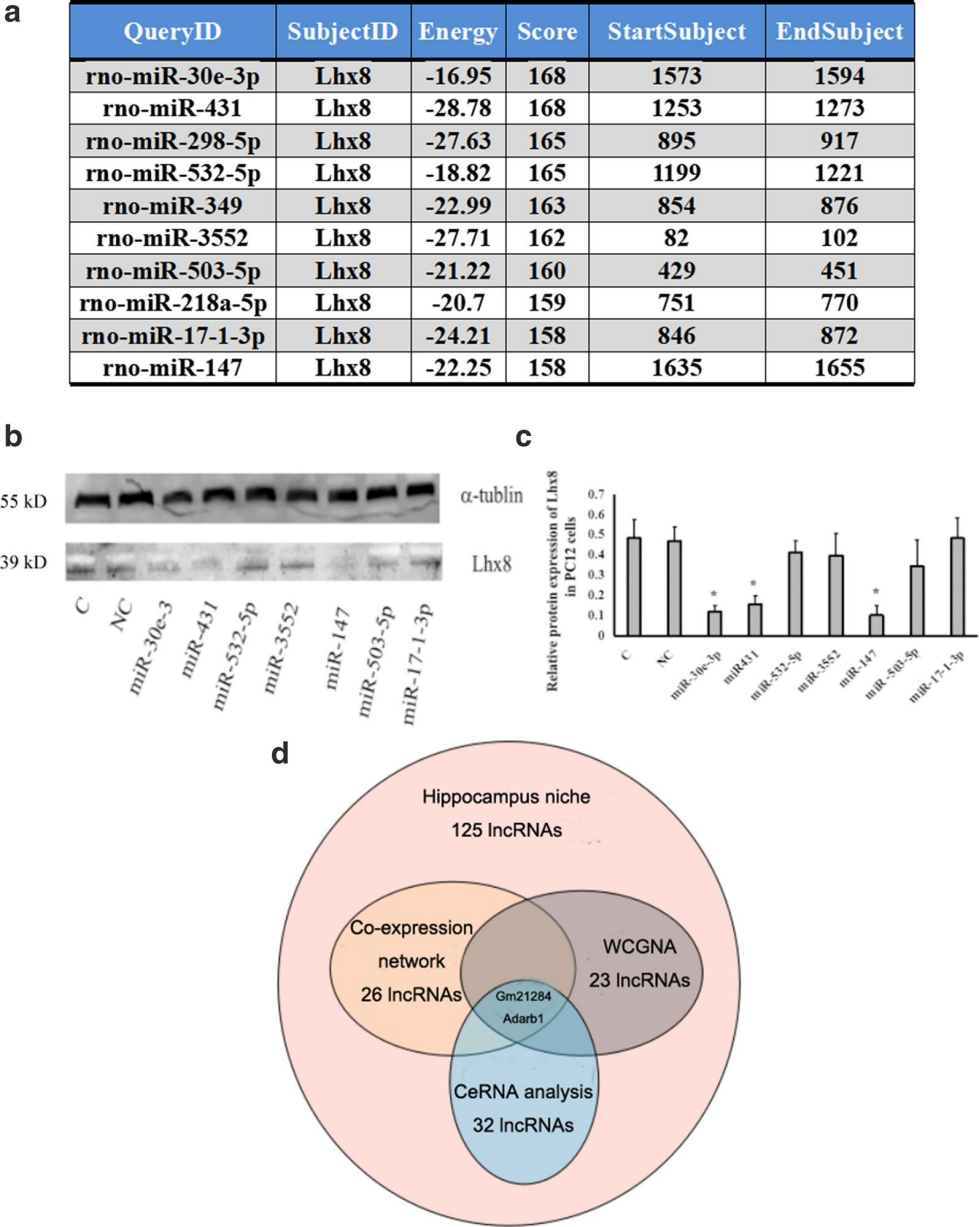
Identification of differential lncRNAs associated with Lhx8 by ceRNA strategy. **a** Prediction of top 10 miRNAs targeting on 3′UTR of Lhx8 by miRanda. **b**, **c** Western blots showed lower expression of Lhx8 in miRNA cells and mimics transfected LV-Lhx8 PC12 cells compared to empty vector infected cells. The difference in Lhx8 expression level between the two groups was significant (*P *< 0.05). **d** Selection and determination of Lhx8 associated lncRNAs. Colored circles represent various lncRNAs: red circle represents the 125 lncRNAs up-regulated in the Gene Chip; yellow circle represents the co-expression of 26 lncRNAs associated with Lhx8 in the co-expression analysis network; brown circle represents the 23 lncRNAs with the same expression module of Lhx8 in WGCNA; blue circle represents 32 lncRNAs predicted by miRanda, which is associated with miR-30e-3P, miR-431, and miR-503-5p, 2 lncRNAs were confirmed after intersection

The CeRNA strategy was applied to filter lncRNAs associated with Lhx8 from the 125 up-regulated lncRNAs in the denervated hippocampus. miRanda predicted the top 10 miRNA candidates for targeting Lhx8 3′ UTR (Fig. 2a). The mimics of the top 10 miRNA candidates were transfected into LV-Lhx8 PC12 which is stable overexpression of Lhx8. Western blotting revealed that the mimics of miR-30e-3p, miR-431, and miR-147 could down-regulate the expression of Lhx8 in LV-Lhx8 PC12, suggesting their participation in the post-transcriptional regulation of Lhx8 expression (Fig. 2b, c). The 125 up-regulated lncRNAs in the denervated hippocampal microenvironment were used as input for miRanda for the prediction of interacting miRNAs. Overall, 17, 22, and 14 lncRNAs were capable of interacting with miR-30e-3P, miR-431, and miR-503-5P, respectively. Among the 32 lncRNAs, differentially expressed in denervated hippocampal niche and capable of interacting with miR-30e-3p, miR-431, and miR-147, 2 (Gm21284 and Adarb1) could be identified after interleaving with lncRNAs in a co-expression network and WGCNA, respectively (Fig. 2d). Gm21284, which has been studied before, was chosen for further experiments.

## 3. Expression and location of Gm21284

**Fig. 3:**
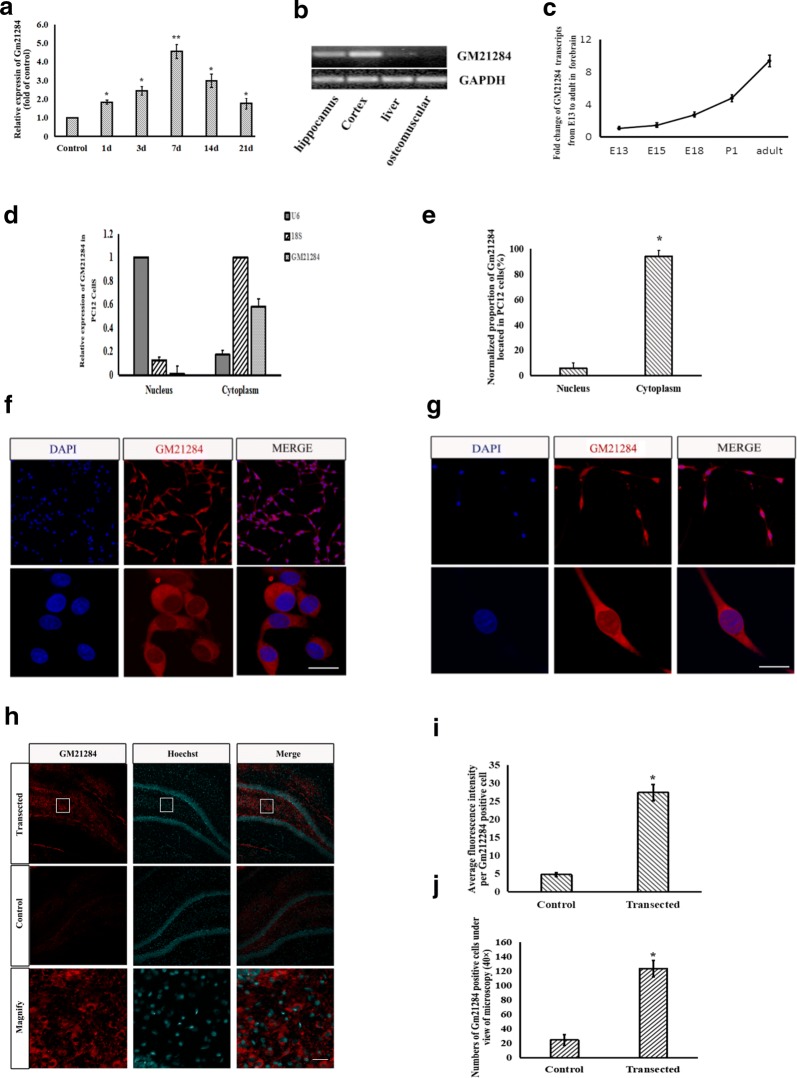
Expression and location of Gm21284. **a** RT-PCR detected Gm21284 expression in hippocampal niche after fimbria–fornix transection at different time points, *: vs. control group, *P *< 0.05; **: vs. control group, *P *< 0.01. **b** RT-PCR detected Gm21284 in tissues originated from different germinal layer. **c** RT-PCR detected Gm21284 in forebrain cortices of E13, E15, E18, and P1 and in adult rats. **d**, **e** RT-PCR showed that the expression of Gm21284 in cytoplasm of PC12 cells was higher than that in nucleus, *: vs. nucleus, *P *< 0.05. **f** FISH assay showed Gm21284 mainly localized in cytoplasm of PC12 cells. **g** FISH assay showed Gm21284 localized mainly in the cytoplasm of hippocampal neurons. **h** FISH labeled Gm21284 positive cells in the dentate gyrus of the hippocampus. **i** FISH showed that the average fluorescence intensity of Gm21284 positive cells in transected side was higher than the control side in DG after fimbria–fornix transection at 7 days, *: vs. control group, *P *< 0.05. **j** Confocal microscopy (×40) showed that the number of Gm21284 positive cells on the transected side was higher than the control side in DG after fimbria–fornix transection at 7 days, *: vs. control group, *P *< 0.05. Scale bar = 50 μm

Hippocampal RNA was isolated at 1, 3, 7, 14, and 21 days after cholinergic innervation. RT-PCR revealed a gradually increase in the expression of Gm21284 after decholinergic innervation and expression reached the highest level at 7 days, which was similar to the expression pattern of Lhx8 observed after denervated innervation in a previous study (Fig. 3a). Unlike protein coding genes, lncRNAs often exhibit its tissue specificity. RT-PCR, which was used to detect the expression of Gm21284 of ectoderm, mesoderm, and endoderm tissues revealed that Gm21284 was expressed only in the cortex and hippocampus derived from the ectoderm and not in the mesoderm-derived skeletal muscle and endodermal-derived liver (Fig. 3b). Genome-wide analysis also showed that lncRNAs possess a dynamic and strictly controlled temporal expression pattern. Gm21284 expression, which represented the different rat developmental stages was observed on E13, E15, E18, and post-natal day (P) 1, and adult cerebral cortex were detected. RT-PCR showed a gradual increase in the expression level of Gm21284 during rat brain development, and highest expression was found in the adult rat cortex; this expression was significantly different from other developmental stages (Fig. 3c). RT-PCR also showed that Gm21284 was mainly expressed in neurons and only rarely in NSCs and astrocytes and that the proportion of expression of Gm21284 in the cytoplasm of PC12 was 94.3% ± 5.7%, whereas that in the nucleus was 5.7% ± 4.3% (Fig. 3d, e). FISH revealed that Gm21284 was mainly located in the cytoplasm of PC12, as well as in cultured hippocampal neurons (Fig. 3f, g). Taken together, Gm21284 can be suggested to participate in the regulation of gene expression mainly through post-transcriptional regulation. The expression and localization of Gm21284 in the hippocampus was further detected by FISH at day 7 after denervation of cholinergic projecting fibers. The hybridization probe signal of Gm21284 was observed in the granular and the SGZ of the denervated hippocampal niche (Fig. 3h), whereas only a weak signal was observed in the control group. The average fluorescence intensity of a positive cell within DG was 27.4 ± 2.6-fold higher in the transected group than in the control group (Fig. 3i). The average number of positive cells in the 40-fold view of confocal microscope in the transected group was 123.5 ± 11.5, which was significantly higher than that in the control group (24.5 ± 7.8) (Fig. 3j).

## 4. Gm21284 acts as ceRNA to regulate expression of Lhx8 with miR-30e-3P

**Fig. 4:**
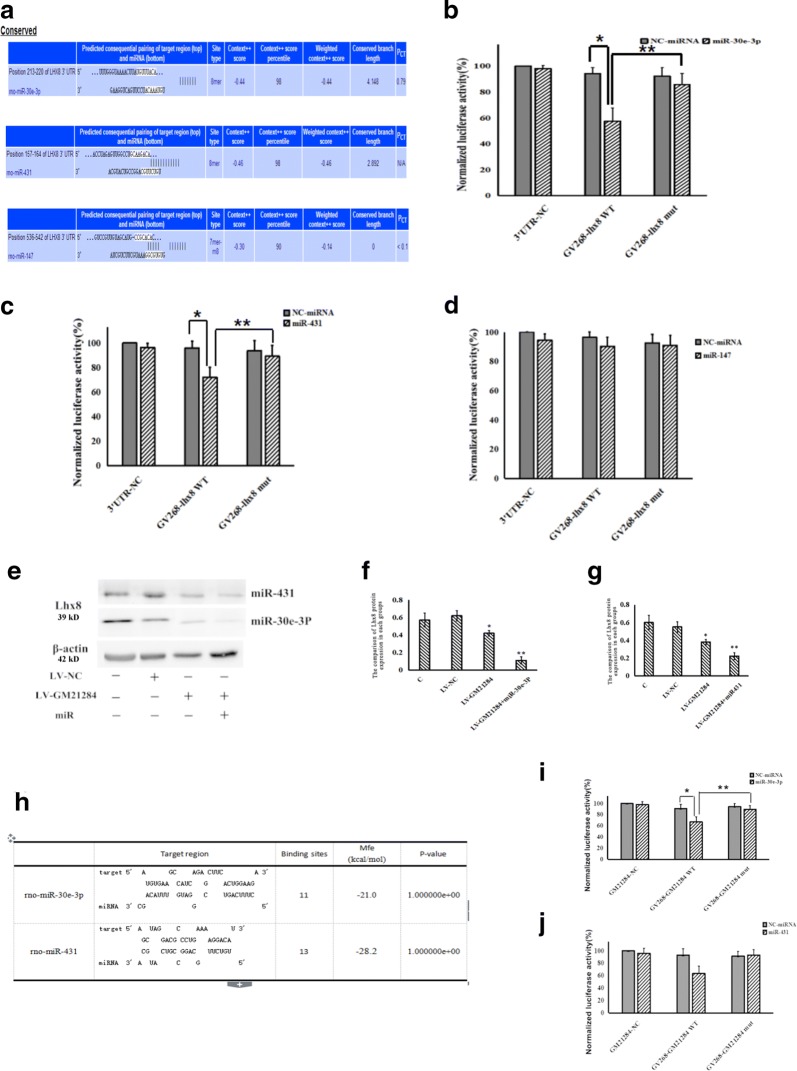
Gm21284 acts as ceRNA to regulate expression of Lhx8 with miR-30e-3P. **a** The predicted target region of miRNAs on Lhx8 3′UTR. **b** Statistical representation of luciferase reporter gene assay for miR-30e-3P, *: vs. NC miRNA, *P *< 0.05; **: vs. GV268-lhx8 mut, *P *< 0.05. **c** Statistical graph of luciferase reporter gene assay for miR-431, *: vs. NC miRNA, *P *< 0.05; **: vs. GV268-lhx8 mut, *P *< 0.05. **d** Statistical graph of luciferase reporter gene assay for miR-147. **e** Relative Lhx8 expression in each group for rescue assay. **f** Statistical graph of rescue assay for miR-30e-3P, *: vs. NC LV-GM21284 + miR-30e-3P group, *P *< 0.05; **: vs. NC group, *P *< 0.05. **g** Statistical graph of rescue assay for miR-431, *: vs. NC LV-GM21284 + miR-431 group, *P *< 0.05; **: vs. NC group, *P *< 0.05. **g** RNAhybrid 2.2 predicted miRNA sponge sites of Gm21284. **h** Statistical graph of luciferase reporter gene assay for miR-30e-3P, *: vs. NC miRNA, *P *< 0.05; **: vs. GV268-lhx8 mut, *P *< 0.05. **i** Statistical graph of luciferase reporter gene assay for miR-431

TargetScan was used to predict the binding sites of miRNAs to the 3′ UTR region of Lhx8, (Fig. 4a). Based on the predicted binding site, a GV268-Lhx8 WT plasmid and a Lhx8 3′UTR mut plasmid were constructed. Dual luciferase reporter assay revealed that the relative luciferase activity was significantly decreased in the GV268-Lhx8 WT + miR-30e-3P mimics group than in the GV268-Lhx8 WT + NC mimics group, and the relative luciferase activity was lower in the GV268-Lhx8 WT + miR-30e-3P mimics group than in the GV268-Lhx8 mut + miR-30e-3P mimics group (Fig. 4b). Dual luciferase reporter assay for miR431 showed similar results with miR-30e-3P (Fig. 4c). The rescue assay suggested that miR-30e-3P and miR431 down-regulates Lhx8 expression, which was up-regulated by the overexpression of Gm21284 in LV-Lhx8 PC12 cells (*P *< 0.05) (Fig. 4e–g).

The dual luciferase reporter assay also revealed that the relative luciferase activity was significantly lower in the GV268-Gm21284 WT + miR-30e-3P group than in the GV268-Gm21284 WT + NC mimics group after addition of miR-30e-3P mimics and in the GV268-Gm21284 mut + miR-30e-3P mimics group (*P *< 0.05), suggesting that Gm21284 binds miR-30e-3P as a sponge (Fig. 4h–j).

### 5. Gm21284 promotes differentiation of NSCs toward cholinergic neurons by competitive inhibition of miR-30e-3P

**Fig. 5:**
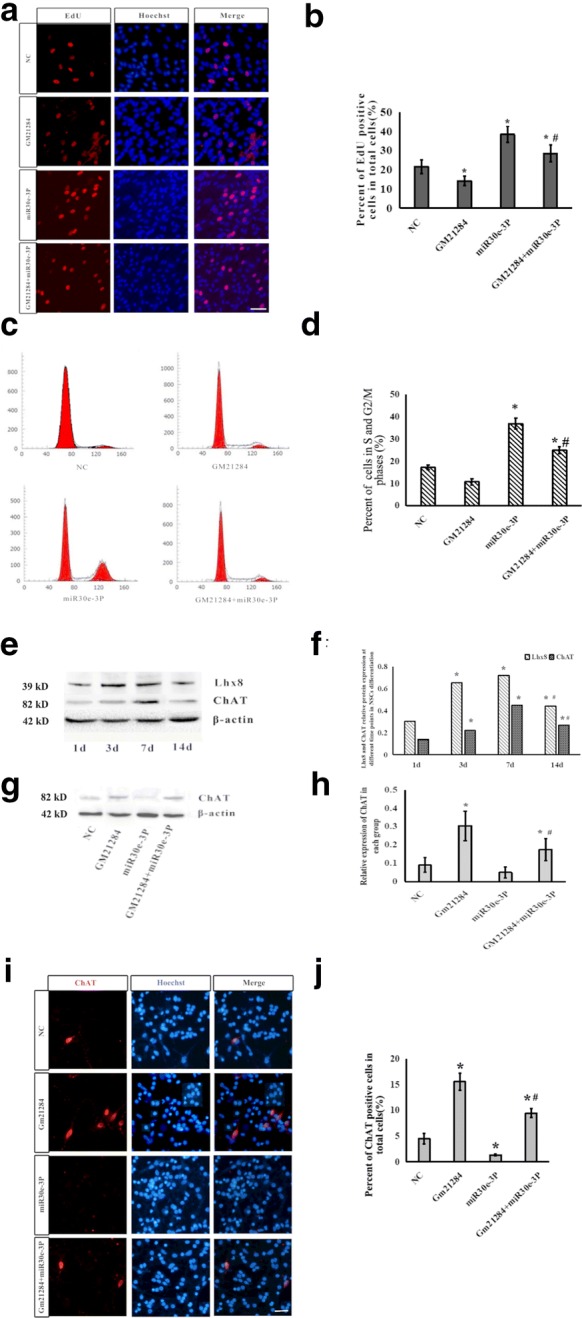
Gm21284 promotes differentiation of NSCs to cholinergic neurons by competitive inhibition of miR-30e-3P. **a** Immunofluorescence images of EdU-positive cells in each group. **b** Statistical graph representing percentage of EdU-positive cells, *: vs. NC group, *P *< 0.05, #: vs. miR30e-3P group, *P *< 0.05. Scale bar = 50 μm. **c** FCS image of cell cycle of hippocampal NSCs in each group. **d** Statistical graph of percentage of cells in the S and G2/M phases, *: vs. NC group, *P *< 0.05, #: vs. miR30e-3P group, *P *< 0.05. **e** Western blot detected the expression of LhX8 and ChAT at different time points in differentiating NSCs. **f** Statistical graph of expression of LhX8 and ChAT, *: vs. 1 day, *P *< 0.05; #: vs. 7 days, *P *< 0.05. **g** Western blot detected the expression of ChAT in each group at 7 days. **h** Statistical graph of expression of ChAT, *: vs. NC group, *P *< 0.05; #: vs. miR30e-3P group, *P *< 0.05. **i** Immunofluorescence images of cells at 7 days after differentiation of NSCs to cholinergic neurons. **j** Statistical graph of percentage of ChAT-positive cells, *: vs. NC group, *P *< 0.05; #: vs. miR30e-3P group, *P *< 0.01. Scale bar = 50 μm

To explore the role of Gm21284 in the development of cholinergic neurons in the hippocampus, we isolated and cultured hippocampal NSCs and divided these into 4 groups: NC, Gm21284, mi-30e-3P, and Gm21284 + miR-30e-3P. The percentage of EdU-positive cells in the Gm21284 group was 14.2% ± 2.5%, which was lower than that in the NC group (21.6% + 3.6%). The percentage of EdU-positive cells in the miR-30e-3P group was 38.4% ± 4.2%, which was higher than that in the NC group (*P* < 0.05). Further, the percentage of EdU-positive cells in the Gm21284 + miR-30e-3P group was higher than that in the NC group, suggesting that the proliferation of neural stem cells was repressed after overexpression in Gm21284 (Fig. 5a, b). Flow cytometry revealed that the percentage of the S- and G2/M-phase cells in NSCs overexpressing Gm21284 decreased significantly. The proportion of neural stem cells in proliferating state could increase after the addition of miR-30e-3P mimics (Fig. 5c, d).

Cellular immunofluorescence was used to detect the differentiation of ChAT-positive cholinergic neurons on 7 days of differentiation of NSCs in the abovementioned four groups. The proportion of ChAT-positive cells in Hoechst-positive cells in the Gm21284 group (15.57% ± 1.64%) was significantly higher than that in the NC group (4.46% ± 1.05%; *P *< 0.05); this proportion was lower in the miR-30e-3P group than in the NC group (1.32% ± 0.2%; *P *< 0.05); ChAT-positive cells in Gm21284 + miR-30e-3P group accounted for Hoechst. The proportion of Hoechst-positive cells (9.45% ± 0.85%) was higher than that in the miR-30e-3P group (*P *< 0.01) (Fig. 5e–j). This suggested that the overexpression of Gm21284 promotes the differentiation of NSCs into cholinergic neurons, whereas miR-30e-3P inhibits the differentiation of NSCs into cholinergic neurons.

## Discussion

The loss of basal forebrain cholinergic neurons and degeneration of hippocampal neurons lead to impairment of learning and memory-related neural circuits. These are also important factors in learning, memory loss, and cognitive dysfunction in patients with Alzheimer’s disease [19–21]. Our previous study found that quiescent NSCs are activated, proliferated, and differentiated into cholinergic neurogenesis after the projection fibers of cholinergic neurons in the basal forebrain are severed. This implies the presence of positive factors in the hippocampal microenvironment that promote neurogenicity after cholinergic innervation [3, 4]. We established a model of cholinergic innervation and detected the expression profile of transcripts in the hippocampal microenvironment. Microarray results indicated a total of 617 differentially expressed mRNAs in the hippocampal microenvironment after decholinergic innervation. KEGG pathway enrichment analysis of the differentially expressed mRNAs indicated that they are mainly involved in signaling pathways, such as at the cholinergic synapses, cell cycle, osteoblast differentiation, and neuronal lineage determination, which confirms a true hippocampal microenvironment after decholinergic innervation. The activation of these signaling pathways is associated with neurogenesis. For example, osteoblast differentiation involves the BMP-SMAD1 pathway, and 10 genes, including BMP and SMAD1, are activated. This pathway is also a classical pathway for stem cell differentiation [22]. Other studies have confirmed that this pathway is associated with embryonic brain development, directed differentiation of stem cells, and neuronal precursor cell migration [22–24].

Numerous studies have suggested the role of lncRNAs in regulating stem cell maintenance and differentiation [25, 26]. For example, specific lncRNAs may be involved in neuronal, epidermal cardiac, endodermal, endothelial, adipocyte, and hematopoietic differentiation [27–32].

Gain of function testing was used to observe the effect of Gm21284 on the proliferation and differentiation of hippocampal NSCs. The proliferation of hippocampal neural stem cells overexpressing Gm21284 and the number of NSCs in the proliferative state decreased. Upon transfection with miR-30e-3P, the proliferative ability of hippocampal NSCs was restored to a certain extent. Upon overexpression of Gm21284 in the hippocampal NSCs, the expression levels of Lhx8 and cholinergic neuronal marker ChAT increased synchronously, reaching the highest level on 7 days, following which these levels decreased. Western blotting and cellular immunofluorescence techniques further confirmed that Gm21284 could promote the differentiation of hippocampal NSCs into cholinergic neurons, whereas miR-30e-3P inhibited this differentiation. Together these results point to a preliminary hypothesis that Gm21284 functions as a ceRNA, which competitively binds to miR-30e-3P and exerts an adsorption-eliminating effect, thereby relieving the negative regulation of Lhx8 gene transcription.

Proliferation and differentiation are two important stages in the development of NSCs, which are mostly present in a resting state in adult brain regions [33, 34]. When the microenvironment undergoes changes, NSCs are activated to undergo symmetrical division, and proliferation produces an increased multipotential for differentiation. Seed cells can also undergo asymmetric division and develop into intermediate progenitor cells. These undifferentiated neuroblasts migrate, mature, and differentiate into differentiation [35, 36]. We observed that the expression level of Gm21284 in the hippocampal neural stem cells was insignificant. The expression of Gm212840 increased gradually with the development of rat forebrain. FISH also confirmed that the major expression of Gm21284 is localized to neurons, which differs from that of the non-coding RNA Otx2c, the product of the Otx2 gene cleavage. No significant difference in the amount of expression in ESCs was observed; however, this expression decreased gradually during neural differentiation, indicating that it plays a role in maintaining the stemness of stem cells [37, 38]. Conversely, the pattern of Gm21284 expression suggests that it plays a role in the establishment or maintenance of specific activities of neuronal subtypes. Conversely, increased expression of Gm21284 in the in vivo cholinergic innervation microenvironment may not be conducive to neurogenesis because it inhibits the proliferation of hippocampal NSCs, prematurely causes NSCs to enter a differentiated state, and reduces the cell pool of NSCs [39, 40]. Therefore, we hypothesize that Gm21284 establishes a threshold for specific target gene activation required for differentiation before the fate of the progenitor cell is determined [32, 41]. Further, it may function as a ceRNA and regulate the expression of Lhx8 in the early stages of cellular fate determination. This is critical for the sustained expression of Lhx8 during differentiation and development of cholinergic neurons. Gm21284 may be important as a promoter of ChAT expression, which functions downstream of Lhx8. However, Gm21284 may also exert an inhibitory effect, which may down-regulate other transcription factors by affecting the chromatin environment or recruiting inhibitory complexes.

## Conclusion

Gm21284 is a long non-coding RNA associated with Lhx8 and expressed differentially in the denervated hippocampus. It acts as a ceRNA and can inhibit the proliferation of hippocampal NSCs and promote their differentiation toward cholinergic neurons by the competitive inhibition of miR-30e-3P.

## Methods and materials

### Animals

A total of 30 adult Sprague–Dawley (SD) rats (17 male and 13 female) and 15 pregnant SD rats (11–19 days) were used in this study and were provided by the Experimental Animal Centre of Nantong University, China. Experimental procedures involving animals were approved by Jiangsu Institutes of Health Guide for the Care and Use of Laboratory Animals. All efforts were made to minimize the number and suffering of animals used in this study. Rats were anesthetized with chloral hydrate (2 ml/kg body weight) before operational procedures. The rat model of cholinergic denervation was performed with a wire knife at the CA1 layer of the dorsal hippocampus at the coordinates of bregma: anteroposterior 1.4 mm; lateral 1–4 mm; depth 5.6 mm. There was no restriction on the sex of the animals.

### Microarray

3 SD rats in each group were used in microarray. Rat hippocampal tissue RNA samples were extracted using TRIzol reagent 7 days after operation. RNA integrity was evaluated by Agilent 2200 Bioanalyzer. The purity of RNA samples was evaluated by ultraviolet spectrophotometer K5500. Fluorescent complementary DNA (cDNA) was synthesized with Amino Allyl MessageAmp II Kit (Life Technologies, USA). RiboArray™lncDETECT™RAT Array 1*12 K (Riobio, China) was applied to detect the transcript profiles of the hippocampus. The slides were scanned and analyzed by GenePix 4000B Microarray Scanner (Molecular Devices, USA). The differentially expressed transcripts were calculated using the Limma package in Bioconductor.

### Real-time polymerase chain reaction (RT-PCR)

3 SD rats in each group were used in RT-PCR for each experiment. Total extracted RNA was quantified and quality checked using Nanodrop 2000 (Thermo Scientific, USA). LncRNAs were reverse transcribed using a RevertAid First Strand cDNA Synthesis Kit (Thermo Scientific, USA) at 65 °C for 5 min, 42 °C for 60 min, and 72 °C for 5 min. The sequence of primers for PCR amplification of Gm21284 as follows: sense 5′-AAGAGACTGTGAGCACCAGGAG-3′ and antisense 5′-TCTCAGCAGAGTCAAGCCATTC-3′, designed and synthesized by RiboBio (Guangdong, China). Quantitative real-time PCR and semi-quantitative PCR were conducted using SYBR Green Master Mix (Roche, Germany) and Dream Taq Green PCR Master Mix (Thermo Scientific), respectively. PCR reactions were performed at 95 °C for 40 s, 59 °C for 40 s, repeated within 40 cycles. GAPDH and U6 were used as endogenous controls. Fold changes were calculated using the relative quantification 2^−∆∆Ct^ method. All experiments were performed in triplicate.

### Hippocampal NSC cell culture

Hippocampal NSCs were isolated from embryos of Sprague–Dawley rats [embryonic day (E) 17]. They were cultured at a density of 1 × 10^5^ cells/ml in an NSC self-renewal medium (DMEM with 2% B27, 20 ng/ml EGF, and 20 ng/ml bFGF). The cells were passaged two generations for PCR, transfection, 5-ethynyl-2′-deoxyuridine (EdU) assay, and flow cytometry assay.

### Fluorescence in situ hybridization (FISH)

FISH was performed in an adult rat coronal brain section in vivo and rat NSCs in vitro using a FISH kit purchased from RiboBio (Guangdong, China) following the manufacturer’s protocol (Additional file 2). After washing the cells twice with PBS, they were fixed in 4% paraformaldehyde for 30 min and incubated in PBS with 0.1% Triton X-100 for 15 min. The hybridization probe was preheated before use. Cells were stained on the second day with Hoechst stain (Sigma, China) after washing twice with 4× saline sodium citrate (SSC) at 42 °C, once with 2× SSC, once with 1× SSC, and once with 1× PBS. Samples were then visualized using a confocal fluorescent microscope (Olympus, Japan). All experiments were performed in triplicate.

### RNA isolation

RNA isolation was performed using a PARIS kit (Ambion, USA). NSCs (1 × 10^7^) were collected and resuspended in 500 µl of ice-cold cell fractionation buffer for 10 min. After centrifugation for 5 min at 4 °C at 500*g*, the supernatant cytoplasmic fraction was carefully collected using a micropipette. Cells in the nuclear fraction pelleted at the bottom of the tube were disrupted by adding 500 µl of ice-cold cell disruption. The cytoplasmic and nuclear lysis/binding solution was mixed and pipetted 3–4 times. To this, 500 µl ACS grade 100% ethanol was added and mixed gently. The sample mixture was drawn through a filter cartridge and washed once with 700 µl wash solution 1 and twice with 500 µl wash solution. Finally, the RNA was eluted with 40–60 µl of elution solution at approximately 95 °C for the RT-PCR assay.

### Transfection

NSCs were transfected using Lipofectamine 2000 (Invitrogen). Plasmid vectors and negative controls for transfection were synthesized using RiboBio. Cells were transfected with a 100 nM plasmid vector using Lipofectamine 2000 according to manufacturer’s instructions. After transfection for 48 h, the expression levels of the selected lncRNAs were measured by quantitative PCR. Cells were collected after 72 h for cell proliferation assays. All experiments were performed in triplicate.

### Cell proliferation assay

Cell proliferation was measured using the EdU (5-ethynyl-2′-deoxyurdine) assay and flow cytometry. For the EdU assay, 1 × 10^5^ cells were suspended in a serum-free DMEM comprising 50 µl of EdU in each group. After incubation in a 1.5-ml centrifuge tube for 2 h, cells were mixed with 4% formaldehyde for 30 min at room temperature. After washing twice with 1 ml PBS, EdU was detected using an Apollo 567 for 30 min at room temperature. Cells were then stained with Hoechst 33342 for 30 min and visualized under a fluorescent microscope (Olympus). The rate of EdU incorporation was expressed as the ratio of EdU-positive cells (red cells) to total Hoechst 33342-positive cells (blue cells). For flow cytometry, cells were fixed overnight at 1 × 10^6^ cells/ml in precooled 75% alcohol after dispersion with trypsin (Sigma) and filtration through a 40-µm cell strainer. Cells were then harvested and stained with Annexin V-fuorescein isothiocyanate and propidium iodide (PI) (BD Biosciences, USA) for 30 min following manufacturer’s instructions. Cells were analyzed using flow cytometry (BD Biosciences, USA). Data were analyzed using the CELL Quest 3.0 software. All experiments were performed in triplicate.

#### Western blot

Lysates of hippocampi and cell from each group in all experiments were separated on 10% SDS-polyacrylamide gels and then transferred to nitrocellulose membrane for identification of Lhx8 and ChAT. Membrane was blocked in TBST containing 5% nonfat milk for 1 h (TBST 100 mM NaCl, 10 mM Tris, 0.05% Tween 20, and pH7.5) and incubated with rabbit anti-Lhx8 (1:200 sigma) and mouse anti-β-actin (1:10,000 Sigma) antibodies in TBST/5% BSA overnight. After washing, membrane was incubated for 2 h with goat peroxidase-conjugated IgG (diluted 1:5000 in TBST/5% BSA). The immunoreactive bands were visualized by SuperSignal West PicoV R Chemiluminescent substrate (Thermo Fisher Scientific Inc.) and visualized with ChemiDocTM Touch Imaging System (Bio-Rad).

### Statistical analysis

Each experiment was performed in three independent biological replicates. All values were evaluated using the SPSS 18.0 statistical software (SPSS, IL, USA) and expressed as mean ± standard deviation. Statistical significance was calculated using the Student’s t-test. *P* value < 0.05 was considered -statistically significant.

## Supplementary information

**Additional file 1.** The detailed results of Microarray.

**Additional file 2.** The Statement of RiboBio.

## Data Availability

The data that support the findings of this study are available.

## Abbreviations

BDNF: brain derived neurophic factor
bFGF: basic fibroblast growth factor
Brn-4: brain-4
ceRNA: competing endogenous RNAs
CNS: central nervous system
EdU: 5-ethynyl-2′-deoxyuridine
EGF: epidermal growth factor
eRNAs: enhancer-associated RNAs
ESTs: expressed sequence tags
FISH: fluorescence in situ hybridization
GDNF: glial cell-derived neurotrophic factor
HARs: human accelerated regions
Lhx8: LIM homeobox 8
lincRNA: long intergenic non-coding RNA
lncRNA: long non-coding RNA
LSD1: lysinespecific demethylase 1
Mash1: mammalian achaete homologue-1
miRNA: microRNA
MRE: miRNA response elements
NGF: nerve growth factor
Ngn2: neurogenin-2 protein
NSCs: neural stem cells
PBS: phosphate buffered saline
PRC2: polycomb repressive complex 2
